# Elevated Circulating Levels of Gut Microbe-Derived Trimethylamine *N*-Oxide Are Associated with Systemic Sclerosis

**DOI:** 10.3390/jcm13195984

**Published:** 2024-10-08

**Authors:** Karen J. Ho, Lutfiyya N. Muhammad, Linh Ngo Khanh, Xinmin S. Li, Mary Carns, Kathleen Aren, Seok-Jo Kim, Priyanka Verma, Stanley L. Hazen, John Varga

**Affiliations:** 1 Department of Surgery, Feinberg School of Medicine, Northwestern University, Chicago, IL 60611, USA; kho1@nm.org; 2 Department of Preventive Medicine, Feinberg School of Medicine, Northwestern University, Chicago, IL 60611, USA; lutfiyya.muhammad@northwestern.edu; 3 Department of Cardiovascular Surgery, Houston Methodist Hospital, Houston, TX 77030, USA; nkjason3003@gmail.com; 4 Department of Cardiovascular & Metabolic Sciences, Lerner Research Institute, Cleveland Clinic, Cleveland, OH 44195, USA; lix2@ccf.org (X.S.L.); hazens@ccf.org (S.L.H.); 5 Department of Medicine, Feinberg School of Medicine, Northwestern University, Chicago, IL 60611, USA; m-carns@northwestern.edu (M.C.); kathleen.aren@northwestern.edu (K.A.); 6 Institute of Basic Science, Sungkyunkwan University, Suwon 16419, Republic of Korea; jokimsteam@gmail.com; 7 Mondrian AI Co., Ltd., Incheon 21985, Republic of Korea; 8 Department of Internal Medicine, University of Michigan Medical School, Ann Arbor, MI 48109, USA; prve@med.umich.edu; 9 Department of Cardiovascular Medicine, Heart, Vascular and Thoracic Institute, Cleveland Clinic, Cleveland, OH 44195, USA

**Keywords:** scleroderma, sclerosis, systemic, trimethylamine *N*-oxide, gastrointestinal microbiome, choline, cluster analysis, gamma-butyrobetaine, choline, case-control studies

## Abstract

**Background/Objectives:** Alterations in fecal microbial communities in patients with systemic sclerosis (SSc) are common, but the clinical significance of this observation is poorly understood. Gut microbial production of trimethylamine (TMA), and its conversion by the host to trimethylamine *N*-oxide (TMAO), has clinical and mechanistic links to cardiovascular and renal diseases. Direct provision of TMAO has been shown to promote fibrosis and vascular injury, hallmarks of SSc. We sought to determine levels of TMAO and related metabolites in SSc patients and investigate associations between the metabolite levels with disease features. **Methods:** This is an observational case:control study. Adults with SSc (*n* = 200) and non-SSc controls (*n* = 400) were matched for age, sex, indices of renal function, diabetes mellitus, and cardiovascular disease. Serum TMAO, choline, betaine, carnitine, γ-butyrobetaine, and crotonobetaine were measured using stable isotope dilution liquid chromatography tandem mass spectrometry. **Results:** Median TMAO concentration was higher (*p* = 0.020) in SSc patients (3.31 [interquartile range 2.18, 5.23] µM) relative to controls (2.85 [IQR 1.88, 4.54] µM). TMAO was highest among obese and male SSc participants compared to all other groups. Following adjustment for sex, BMI, age, race, and eGFR in a quantile regression model, elevated TMAO levels remained associated with SSc at each quantile of TMAO. **Conclusions:** Patients with SSc have increased circulating levels of TMAO independent of comorbidities including age, sex, renal function, diabetes mellitus, and cardiovascular disease. As a potentially modifiable factor, further studies examining the link between TMAO and SSc disease severity and course are warranted.

## 1. Introduction

Systemic sclerosis (SSc) is a chronic disease of unknown etiology characterized by fibrosis, vasculopathy, and immune dysregulation [1]. Progressive fibrosis leads to disability, organ failure, and death [1]. The diagnosis of SSc can be challenging due to low prevalence, the non-specific nature of early symptoms, and heterogeneity in clinical presentation. Current classifications are based on the degree of skin involvement, specificity of autoantibodies, and major organ-based complications [2]. However, these schemata do not completely capture the full spectrum in clinical manifestations nor provide precise prognostication of disease trajectories and response to therapy [3]. Thus, there is an unmet need to identify exposures and variables that modulate the pathogenesis of SSc and may facilitate diagnosis and assessment of disease progression and response to therapy.

It has been noted that the structure and composition of fecal commensal communities in patients with SSc relative to healthy individuals is substantially altered, a finding that has been described in over 75% of patients with SSc, particularly those with significant esophageal dysmotility [4,5]. Importantly, patients with SSc have significantly lower levels of commensal genera deemed to be protective against inflammation, such as *Bacteroides*, *Faecalibacterium*, and *Clostridium*, and significantly higher levels of pathobiont genera, such as *Fusobacterium*, compared with non-SSc controls [6,7]. Abundance of *Clostridium* is inversely associated with severity of gastrointestinal symptoms in both cohorts [6]. However, whether alterations in fecal microbial communities in these patients is a causative factor in the development or progression of disease or is secondary to intestinal dysmotility, dietary alterations, antibiotic therapy and other medications used to treat SSc, remains unknown [8].

The gut microbiome produces trimethylamine, which can be absorbed into the host circulation and converted to trimethylamine *N*-oxide (TMAO) through the action of the hepatic flavin-containing enzyme monooxygenase 3 (FMO3) [9,10,11]. Diets rich in choline, carnitine, and lecithin are associated with high circulating levels of TMAO [12,13,14]. Recent studies have identified a receptor for TMAO, the protein kinase R-like endoplasmic reticulum kinase (PERK) [15]. We recently demonstrated that incubation of explanted normal human fibroblasts or microvascular endothelial cells with TMAO induced their activation and transformation into profibrotic myofibroblasts [16]. Elevated TMAO has been associated with experimental and human fibrosis. In particular, TMAO has been shown to induce renal [17,18] and myocardial fibrosis in disease models [19,20,21,22]. In large cohort studies, elevated TMAO is associated with increased mortality risk in cardiovascular disease [23] and left ventricular dysfunction in heart failure [24]. A recent publication found elevated levels of TMAO in adults with SSc compared to non-SSc controls [25]. However, despite these intriguing observations, the pathogenic contribution and precise mechanisms of TMAO-induced fibrosis have not been clearly elucidated within the context of SSc.

Considering the experimental evidence linking TMAO with fibrosis and vascular injury and the observed associations of elevated TMAO and fibrotic and vascular diseases, we explored the involvement of TMAO in SSc, a disease characterized by vascular injury and fibrosis. In particular, we sought to (1) compare serum TMAO levels in patients with SSc from a large well-annotated registry to age- and sex-matched non-SSc controls; and (2) investigate associations between serum TMAO levels with clinical features and complications of SSc. Investigating the relationship between serum TMAO with SSc could lead to novel insights into the potential interactions of environmental factors, such as dietary components, with host physiology in the development and progression of SSc.

## 2. Materials and Methods

### 2.1. Study Design, Data Source, and Study Population

In this observational nested case-control study [26], 200 participants with SSc and age ≥ 18 years were selected from the Northwestern Scleroderma Program Registry and Biorepository, which collects anthropometric, demographic, and medication data in a standardized and prospective manner [27]. Participants met the 2013 American College of Rheumatology/European League Against Rheumatism classification criteria for SSc [28]. Longitudinal clinical data (baseline and 1 year follow-up) for forced vital capacity (FVC), diffusing capacity for carbon monoxide (DLCO), and modified Rodnan skin score (MRSS) are included in the Northwestern Scleroderma Program Registry and Biorepository. Obesity was defined as a body mass index (BMI) greater than or equal to 30 kg/m^2^. Cardiovascular disease was defined by a history of myocardial infarction, stroke or transient ischemic attack, or coronary revascularization (bypass graft or percutaneous procedure). Participants were selected in a consecutive fashion based on availability of adequate (≥ 50 microliters) serum samples that had not been previously thawed (i.e., remained frozen since the time of sample collection) and presence of all relevant clinical data. The “baseline” time point refers to the time at entry into the biorepository, which also corresponds to the time of serum sampling. One source of the control group without SSc (*n* = 340) was the Cleveland Clinic GeneBank [29,30,31], a research repository comprised of sequential consenting stable subjects undergoing elective cardiac evaluation and who were subsequently followed longitudinally for incident cardiovascular disease outcomes. A second source of controls (*n* = 60) was BioBank, which consists of subjects undergoing cardiac risk factor evaluation/modification in a preventive cardiology clinic [30,32,33]. These controls were selected based on the following matching criteria with the SSc group: age (within ±2 years of the participants with SSc), sex, estimated glomerular filtration rate (eGFR; CKD-EPI), type 2 diabetes mellitus, and cardiovascular disease. Informed consent was obtained from all participants. This study protocol was approved by the Northwestern University (IRB STU00211009, approved 27 September 2020) and the Cleveland Clinic (IRB 7338, approved 17 September 2023 and IRB 4265, approved 9 March 2024) Institutional Review Boards.

### 2.2. Metabolomics

Serum samples from the SSc group (50 microliters per sample) were thawed and processed in a single batch. TMAO was measured using stable isotope dilution liquid chromatography tandem mass spectrometry as previously described [34]. Serum TMAO in the control group had been previously measured for a separate study [28]. Within the SSc group, concentrations of choline, betaine, carnitine, γ-butyrobetaine, and crotonobetaine were also measured using stable isotope dilution liquid chromatography tandem mass spectrometry by established methods [13]; concentrations of these additional metabolites were not available in the control group.

### 2.3. Statistical Analysis

Baseline demographics and clinical characteristics are reported using summary statistics. Demographics and clinical characteristics at baseline were compared using Wilcoxon rank sum and Fisher’s exact test. Wilcoxon rank sum test was used to determine whether baseline serum TMAO concentration could distinguish participants with SSc from healthy controls. Distributions of baseline serum TMAO concentrations by SSc and control groups are graphically displayed using box plots. Box plots were also created using the natural log (ln) of TMAO. The Van Elteren test, an extension of the Wilcoxon rank sum for stratified analyses, was utilized to assess whether obesity and sex are confounders in the relationship between TMAO and SSc. Since there were three TMAO observations (one in the SSc group and two in the control group) that were outliers, we reported results with and without these outliers. Specifically, the box plots, Wilcoxon rank sum test results, and Van Elteren test results are reported with and without outliers. Quantile regression was fitted with TMAO as the outcome. Covariates in the quantile regression model included sex, group (SSc vs. control), BMI, age, race, and eGFR. Quantile regression coefficients, 95% confidence intervals (CI), and corresponding *p* values are reported to summarize the model findings. The estimated quantile regression coefficients of the group variable and 95% CI are plotted by TMAO quantile levels to illustrate TMAO differences between the SSc and control groups when adjusting for sex, BMI, age, race, and eGFR. The estimated coefficients from the 25th and 50th quantiles were compared with the 75th estimated quantile regression coefficients using an F-test to determine if there were significant differences. In an exploratory analysis, a heat map of serum metabolite concentrations was constructed to identify hierarchical clusters among the participants with SSc. The complete linkage clustering algorithm with Euclidean distances was used in the heat map to produce clusters based on the serum metabolite concentrations among the participants with SSc. A dendrogram is included in the heat map to display the arrangement of hierarchical clusters. Serum metabolite concentrations that were included in the exploratory analysis were betaine, γ-butyrobetaine, carnitine, choline, crotonobetaine, and TMAO. For the exploratory analysis, we did not remove the outlier observation (i.e., no sensitivity analysis was conducted for the exploratory analysis). Comparisons are made between the cluster groups identified through the heat map for continuous and categorical baseline demographics and clinical characteristics. For continuous variables, *p* values for the Wilcoxon rank sum test or Kruskal—Wallis test are reported. Chi-square test or Fisher’s exact test *p* values are reported for categorical variables. Spearman correlations and corresponding *p* values were computed to assess associations between serum metabolite concentrations and clinical factors. *p* values were adjusted using the Benjamini—Hochberg procedure for the exploratory analyses that tested the relationship among the serum metabolite concentrations and clinical factors such as disease duration, disease progression over 1 year, and antibodies. A *p* value < 0.05 was considered statistically significant. All statistical analyses were conducted in R version 4.2.2.

## 3. Results

Baseline demographics and clinical characteristics of the study participants are summarized in Table 1. The SSc group included fewer males than females and participants predominantly identified as Caucasians. BMI was significantly different between the SSc and control groups, with higher median BMI values in the latter group (*p* < 0.001).

**Figure 1:**
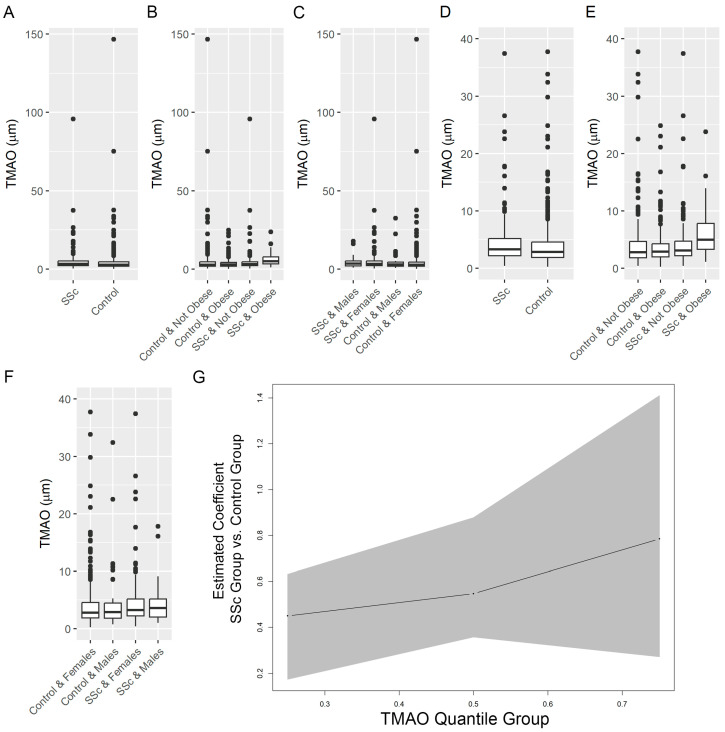
Comparative analysis of serum TMAO concentration. Each comparison is shown with and without outlier observations. For the comparison of control and SSc groups, box plots of TMAO in the control and SSc groups is shown with (**A**) and without (**D**) the outlier observations in both groups. For the comparison based on SSc group and obesity, box plots of TMAO in each group are shown with (**B**) and without (**E**) the outlier observations in the non-obese groups (2 in control group and 1 in SSc group). For the comparison based on SSc group and sex, box plots of TMAO in each group are shown with (**C**) and without (**F**) the outlier observations in the female groups (two in control group and one in SSc group). Data shown are median with interquartile range. (**G**) Quantile regression analysis of the differences in TMAO at each quantile between the SSc and control groups. After adjusting for sex, BMI, age, race, and eGFR, SSc participants had an elevated TMAO level in comparison to control participants at all quantile of TMAO. Quantile regression estimated coefficients at each TMAO quantile are shown in Table 1.

As shown in Figure 1A, the median serum TMAO concentration of 3.31 µM (IQR: [2.18, 5.23]) in the SSc group was significantly higher than in the control group (2.85 µM; IQR [1.88, 4.54]; *p* = 0.020). In one SSc participant, TMAO level was markedly higher (95.71 µM) than the other TMAO observations. Two control participants had values that were considered outliers (146.58 µM and 75.16 µM). The difference in TMAO between the SSc and control groups remained statistically significant even after exclusion of the outliers (Figure 1D), (SSc group median = 3.29 µM, IQR: [2.18, 5.18]; control group median = 2.83 µM, IQR: [1.87, 4.54]; *p* = 0.019). The distribution of TMAO for each group was also examined using the natural log of TMAO concentration (Supplementary Figure S1). All statistical results using the natural log of TMAO concentration were consistent with the original TMAO results.

There were statistically significant differences in serum TMAO concentration based on SSc group and obesity (*p* = 0.007, Van Elteren). Obese SSc participants (*n* = 38) had the highest median serum TMAO concentration (4.94 µM (IQR: [3.30, 7.79]), followed by non-obese SSc (*n* = 162; 3.10 µM (IQR: [2.15, 4.71]), obese control (*n* = 172; 2.90 µM (IQR: [1.94, 4.24]), and non-obese control participants (*n* = 228; 2.80 µM (IQR: [1.83, 4.54]). Box plots of TMAO concentrations in these 4 groups with and without the TMAO outliers in the non-obese control and SSc groups are shown in Figure 1B,E, respectively.

Male SSc participants had the highest median serum TMAO concentration (*n* = 31; 3.61 µM (IQR: [2.00, 5.17]), followed by female SSc (*n* = 169; 3.27 µM (IQR: [2.19, 5.2]), male control (*n* = 71; 2.88 µM (IQR: [1.80, 4.46]) and female control participants (*n* = 329; 2.81 µM (IQR: [1.89, 4.54]) (*p* = 0.019, Van Elteren). Box plots of TMAO concentrations in these four groups with and without the TMAO outliers in the female control and SSc groups are shown in Figure 1C,F, respectively.

Quantile regression estimated coefficients at each TMAO quantile are shown in Supplementary Table S1. Figure 1G illustrates the differences in TMAO at each quantile between the SSc and control groups from the quantile regression model. After adjusting for sex, BMI, age, race, and eGFR, SSc participants had an elevated TMAO level compared to control participants at all quantiles of TMAO. eGFR was also significantly associated with TMAO in the adjusted quantile regression models. Statistically significant differences were found when comparing the estimated 25th quantile coefficients to the estimated 75th quantile coefficients (F = 5.21, *p* <0.001) and comparing the estimated 50th quantile coefficients with the estimated 75th quantile coefficients (F = 3.29, *p* = 0.003).

Bivariate associations of metabolite concentrations with participant characteristics in the SSc cohort were performed. TMAO is inversely associated with DLCO at baseline and positively associated with change in DLCO over one year, respectively (Supplementary Table S2A), crotonobetaine is higher in the diffuse cutaneous class of SSc compared to limited cutaneous (Supplementary Tables S2B), betaine is lower in patients with anti-topoisomerase-1 antibody (Supplementary Table S2C), and TMAO is higher in patients with anti-centromere antibody (Supplementary Table S2E). These associations were not statistically significant when the *p* values were adjusted using the Benjamini-Hochberg procedure. No significant correlations between TMAO concentration and MRSS, presence of pulmonary hypertension or interstitial lung disease, and change in MRSS and FVC over 1 year, were observed (Supplementary Table S2A,G).

**Figure 2:**
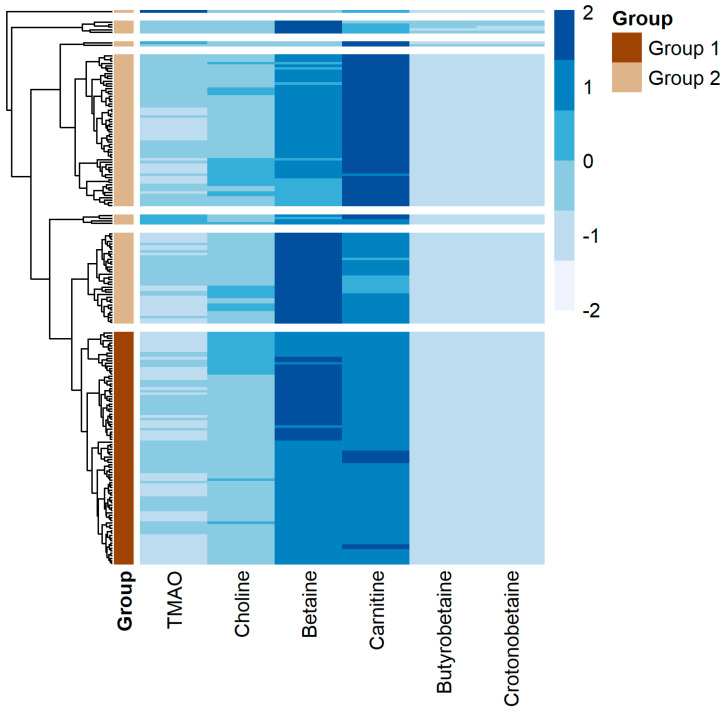
Heat map of serum metabolite concentrations in the SSc cohort. Metabolites are represented in the columns and individual participants are represented in the rows. As described in the text, 7 hierarchical clusters were initially identified and then regrouped into 2 clusters based on sample sizes. The final 2 groups are indicated on the left side of the figure by dark brown (*n* = 92) or tan (*n* = 108).

In an exploratory analysis, hierarchical clustering of the six TMAO-related metabolites in the SSc group was performed. Due to the small sample sizes within the hierarchical clusters, the smallest 6 clusters were combined into one group. The demographic and clinical characteristics and metabolite concentrations of the 2 groups are shown (Figure 2, Supplementary Table S3). Group 1 consisted of older participants, more males, and more participants diagnosed with diffuse cutaneous systemic sclerosis in comparison to group 2. Additionally, participants in group 1 had higher medians for all serum metabolite concentrations except crotonobetaine, and higher baseline FVC and DLCO.

## 4. Discussion

Herein, we found that serum TMAO levels were elevated in SSc patients compared to non-SSc controls matched for age, sex, renal function, and history of diabetes mellitus and cardiovascular disease. TMAO elevation was more pronounced in male and in obese SSc participants. We did not find significant associations between TMAO or related metabolites with clinical markers of SSc severity, disease subtype, presence of interstitial lung disease, or longitudinal change in pulmonary function or MRSS over 1 year.

### 4.1. Involvement of TMAO in SSc

Gut microbiota are responsible for the production of TMA, the metabolic precursor of TMAO [29]. Recent studies have demonstrated elevated TMAO in chronic cardiovascular diseases, renal insufficiency, obesity, and other conditions [17,18,19,20,24,35]. Moreover, TMAO has been shown to trigger vascular injury and fibrosis [16,36], suggesting that elevated TMAO might play a direct pathogenic role in these diseases. Gut dysbiosis has been described in patients with SSc [6,7,37], and fibrosis and vascular injury are both prominent hallmarks of both limited and diffuse cutaneous forms of SSc [38]. These observations indicate that alterations in the gut microbiota may have a role in SSc, possibly via the production of mediators. Indeed, we recently showed that TMAO was a potent inducer of fibroblast activation, and the transdifferentiation of both fibroblasts and endothelial cells into profibrotic myofibroblasts [16]. The pro-fibrotic effects of TMAO and the molecular pathways of these effects in the heart, kidney, liver, and skin have recently been reviewed [36]. However, the involvement of TMAO in SSc has not been widely explored in humans. We now report that TMAO is elevated in adults with SSc, consistent with a recent report by Stec et al. [25] using a smaller cohort (*n* = 110) of adults with SSc and healthy controls. The median plasma levels of TMAO in SSc patients (283.0 ng/mL, equivalent to 3.76 mM) and controls (205.5 ng/mL, equivalent to 2.74 mM) in the Stec et al. report are comparable to the serum concentrations observed in the current study. We paradoxically did not observe an association between TMAO and MRSS or ILD, suggesting a likely complex relationship between TMAO and disease features that need further investigation in a larger cohort. The lack of these correlations suggests that there may be factors unaccounted for that influence the relationship between SSc and TMAO or that serum TMAO levels do not fully reflect the activity of TMAO at the tissue level.

### 4.2. Sex-Related Differences in TMAO Levels

There are distinctive sex-related differences in TMAO levels. In both rodents and humans, TMAO is higher in females [39,40,41] due to the higher hepatic concentration of FMO3, which converts TMA to TMAO, in females. In humans, TMAO levels have been found to be higher during menstruation and menopause [42], while in men, testosterone is associated with the downregulation of FMO3 expression [10], which may account for the higher ratio of TMA to TMAO in men. Thus, our finding that TMAO is higher in male control SSc than female SSc participants was unexpected and warrants further investigation.

### 4.3. TMAO and Obesity

While the associations of TMAO with obesity and sex in SSc cohorts have not been previously reported, others have also found TMAO correlations with BMI in humans [43], and with obesity traits in mice [29]. Mice with Western diet-induced obesity had higher plasma TMAO levels, body weight, dyslipidemia, and cardiac dysfunction compared to mice on a normal diet. However, when mice were fed a Western diet while being treated with an inhibitor of TMA formation (3,3-Dimethyl-1-butanol [DMB], DMB treatment had no effects on body weight and dyslipidemia, but significantly reduced TMAO levels and prevented cardiac dysfunction [44]. Among children, TMAO is positively associated with BMI, waist circumference and peripheral blood pressure but is not associated with the Homeostatic Model Assessment of Insulin Resistance (HOMA-IR) or insulin levels [45]. In a cross-sectional observational study of adults, TMAO levels were associated with BMI, visceral adiposity index, and fatty liver index [46]. In the prospective Cardiovascular Health Study of over 4000 older American adults, TMAO level was associated with fasting insulin level but not insulin sensitivity or risk of type 2 diabetes mellitus [47]. Whether TMAO impacts the disease course of SSc and other fibrotic diseases in people with obesity is currently unknown and is an interesting area for future discussion.

### 4.4. TMAO-Related Metabolites and SSc

Prior studies have not examined associations between TMAO-related metabolites and SSc. Gut microbial metabolism of phosphatidylcholine, the major dietary source of choline, is first converted to TMA followed by host hepatic flavin monooxygenase mediated conversion to TMAO [29]. While choline and carnitine are substrates for microbial TMA lyase, they are not known to be profibrotic or associated with SSc. An alternative TMA nutrient found predominantly in red meat, L-carnitine, is another dietary source for gut microbe-dependent formation of TMAO [13]. γ-Butyrobetaine is the immediate biosynthetic precursor, via crotonobetaine, to carnitine during endogenous carnitine synthesis [48]. Since we did not observe any significant bivariate associations between any of these metabolites with clinical features of SSc, we performed hierarchical clustering was to determine if we could partition the individuals with SSc into groups based on similarities across levels of TMAO and other metabolites. This exploratory analysis suggests that these metabolites identify subsets of SSc patients with distinct phenotypes.

### 4.5. Limitations

The present cohort-nested case-control study has several limitations. First, as we used a single control cohort, it will be important to validate our findings with a larger study and refine the sensitivity and specificity of TMAO as a biomarker for SSc. Notably, the control cohort was a matched non-SSc cohort, but was not a “healthy” cohort as the subjects were either undergoing coronary evaluation or a coronary angiogram for coronary artery disease. Second, TMAO was quantified in the control and experimental cohorts asynchronously. However, metabolomics in both cohorts was performed in a core laboratory using the same validated methodologies. Third, we examined TMAO in a single non-fasting serum sample for each SSc participant, and it is possible that repeated measurements over time will provide a more accurate assessment of TMAO. Furthermore, TMAO is known to have diurnal variation with higher levels in the evening chronotype than the morning chronotype [49]. However, fasting plasma levels of TMAO for a healthy Western population from Europe and America have been reported to be relatively stable over a 12-month period, independent of diet, lifestyle, or routine biochemistry analysis, including blood lipid measurements [50]. Fourth, while the metabolites under investigation in this study vary according to diet [12,13,14], dietary information was not collected for either the control or the SSc group, and hence we were unable to account for dietary determinants of the levels of TMAO and the other metabolites. We also lacked data on medications that are known to affect the gut microbiome, including proton pump inhibitors, antibiotics, immunosuppressants, and probiotics. Fifth, the observational cross-sectional design of this study does not allow us to determine if TMAO is a bioactive modulator of SSc development or progression. Our previous studies demonstrating that TMAO drives fibrogenic mesenchymal transition [16], combined with work of others [17,18,19,20,23,24,40,51]. demonstrating a profibrotic effect of TMAO in multiple organ systems, strongly suggest that TMAO plays a role in SSc pathogenesis. However, the fact that TMAO did not discriminate between SSc severity or phenotypes or longitudinal change over 1 year suggests that there are likely other factors mediating the effect of TMAO in vivo.

## 5. Conclusions

In summary, we provide evidence that high serum levels of TMAO are associated with SSc. Future work should focus on further understanding the mechanisms by which TMAO modifies the development and course of SSc, identifying novel TMAO-based therapeutic strategies and treatment adjuncts, and validating these findings in a larger cohort that prospectively collects microbiome- and SSc-specific exposures.

## Figures and Tables

**Table 1:** Demographics and baseline clinical characteristics.

	Control Group *n* = 400	SSc Group *n* = 200	*p* Value
Age, years (median [IQR])	53.63 [45.86, 62.98]	52.00 [43.75, 60.00]	0.040 *
Female (%)	383 (95.6)	169 (84.5)	0.565
Caucasian (%)	363 (92.8)	165 (82.5)	<0.001 *
BMI (median [IQR])	29.66 [26.01, 34.01]	24.76 [21.35, 28.37]	<0.001 *
eGFR, mL/min/1.73 m^2^ (median [IQR])	93.08 [81.76, 103.82]	92.62 [75.83, 106.06]	0.469
Creatinine, mg/dL (median [IQR])	0.76 [0.67, 0.83]	0.77 [0.68, 0.88]	0.200
History of cardiovascular disease (%)	14 (3.5)	6 (3.2)	1.000
History of diabetes mellitus (%)	12 (3.0)	5 (2.6)	1.000
SSc classification			
Limited cutaneous (%)		87 (43.5)	
Diffuse cutaneous (%)		109 (54.5)	
Stiff skin syndrome (%)		4 (2.0)	
Disease duration, months (median [IQR])		46.00 [17.50, 97.50]	
Autoantibodies			
Anti-nuclear antibody (%)		190 (96.4)	
Anti-centromere antibody (%)		39 (20.1)	
Anti-topoisomerase I antibody (%)		43 (22.4)	
Anti-RNA polymerase III antibody (%)		56 (34.1)	
MRSS (median [IQR])		8.00 [4.00, 18.00]	
FVC, liters (median [IQR])		2.84 [2.45, 3.38]	
DLCO, mL/mm Hg/min (median [IQR])		14.60 [11.10, 18.35]	

BMI, body mass index; DLCO, diffusing capacity of the lungs for carbon monoxide; eGFR, estimated glomerular filtration rate; FVC, forced vital capacity; IQR, interquartile range; MRSS, modified Rodnan skin score; *n*, number; SSc, systemic sclerosis. * Indicates *p* value < 0.05.

## Data Availability

The data that support the findings of this study are available from the corresponding author, John Varga, upon reasonable request, due to patient privacy considerations in accordance with institutional data sharing restrictions.

## Supplementary Materials

The following supporting information can be downloaded at: https://www.mdpi.com/article/10.3390/jcm13195984/s1, Figure S1: Comparative analysis of serum TMAO concentration based on SSc group (A), SSc group and obesity (B), and SSc group and sex (C); Table S1: A. Quantile Regression Parameter Estimates at the 25th Quantile of TMAO, B. 50th Quantile of TMAO, C. and 75th Quantile of TMAO; Table S2: A. Spearman’s Rho Correlations for metabolites and disease duration at baseline and progression over 1 year, B. Kruskal-Wallis Test for Comparing SSc Classification by Metabolites, C. Wilcoxon Rank Sum Test for Comparing Anti-Scl-70 (Anti-Topoisomerase-1 antibody) by Metabolites, D. Wilcoxon Rank Sum Test for Anti-Neutrophil Antibody by Metabolites, E. Wilcoxon Rank Sum Test for Anti-Centomere Antibody by Metabolites. F. Wilcoxon Rank Sum Test for Anti-RNA Polymerase III by Metabolites, G. Wilcoxon Rank Sum Test for Pulmonary Hypertension/Interstitial Lung Disease by Metabolites. Table S3: Characteristics and metabolites of the 2 patient groups identified by hierarchical clustering. *p* values for the Wilcoxon rank sum test are reported for continuous variables. Chi square test or Fisher’s exact test *p* values are reported for categorical variables.

## References

[1] Allanore Y., Simms R., Distler O., Trojanowska M., Pope J., Denton C.P., Varga J. (2015). Systemic sclerosis. Nat. Rev. Dis. Primers.

[2] Denton C.P., Khanna D. (2017). Systemic sclerosis. Lancet.

[3] Varga J., Hinchcliff M. (2014). Connective tissue diseases: Systemic sclerosis: Beyond limited and diffuse subsets?. Nat. Rev. Rheumatol..

[4] Bellocchi C., Volkmann E.R. (2018). Update on the Gastrointestinal Microbiome in Systemic Sclerosis. Curr. Rheumatol. Rep..

[5] Andreasson K., Alrawi Z., Persson A., Jonsson G., Marsal J. (2016). Intestinal dysbiosis is common in systemic sclerosis and associated with gastrointestinal and extraintestinal features of disease. Arthritis Res. Ther..

[6] Volkmann E.R., Hoffmann-Vold A.M., Chang Y.L., Jacobs J.P., Tillisch K., Mayer E.A., Clements P.J., Hov J.R., Kummen M., Midtvedt Ø. (2017). Systemic sclerosis is associated with specific alterations in gastrointestinal microbiota in two independent cohorts. BMJ Open Gastroenterol..

[7] Volkmann E.R., Chang Y.L., Barroso N., Furst D.E., Clements P.J., Gorn A.H., Roth B.E., Conklin J.L., Getzug T., Borneman J. (2016). Association of Systemic Sclerosis with a Unique Colonic Microbial Consortium. Arthritis Rheumatol..

[8] Denton C.P., Murray C. (2019). Cause or effect? Interpreting emerging evidence for dysbiosis in systemic sclerosis. Arthritis Res. Ther..

[9] Seim H., Schulze J., Strack E. (1985). Catabolic pathways for high-dosed L(-)- or D(+)-carnitine in germ-free rats?. Biol. Chem..

[10] Bennett B.J., de Aguiar Vallim T.Q., Wang Z., Shih D.M., Meng Y., Gregory J., Allayee H., Lee R., Graham M., Crooke R. (2013). Trimethylamine-N-oxide, a metabolite associated with atherosclerosis, exhibits complex genetic and dietary regulation. Cell Metab..

[11] Al-Waiz M., Mikov M., Mitchell S.C., Smith R.L. (1992). The exogenous origin of trimethylamine in the mouse. Metab. Clin. Exp..

[12] Wang Z., Bergeron N., Levison B.S., Li X.S., Chiu S., Jia X., Koeth R.A., Li L., Wu Y., Tang W.H.W. (2019). Impact of chronic dietary red meat, white meat, or non-meat protein on trimethylamine N-oxide metabolism and renal excretion in healthy men and women. Eur. Heart J..

[13] Koeth R.A., Wang Z., Levison B.S., Buffa J.A., Org E., Sheehy B.T., Britt E.B., Fu X., Wu Y., Li L. (2013). Intestinal microbiota metabolism of L-carnitine, a nutrient in red meat, promotes atherosclerosis. Nat. Med..

[14] Koeth R.A., Lam-Galvez B.R., Kirsop J., Wang Z., Levison B.S., Gu X., Copeland M.F., Bartlett D., Cody D.B., Dai H.J. (2019). l-Carnitine in omnivorous diets induces an atherogenic gut microbial pathway in humans. J. Clin. Investig..

[15] Chen S., Henderson A., Petriello M.C., Romano K.A., Gearing M., Miao J., Schell M., Sandoval-EspinolaEspinola W.J., Tao J., Sha B. (2019). Trimethylamine N-Oxide Binds and Activates PERK to Promote Metabolic Dysfunction. Cell Metab..

[16] Kim S.J., Bale S., Verma P., Wan Q., Ma F., Gudjonsson J.E., Hazen S.L., Harms P.W., Tsou P.-S., Khanna D. (2022). Gut microbe-derived metabolite trimethylamine N-oxide activates PERK to drive fibrogenic mesenchymal differentiation. iScience.

[17] Gupta N., Buffa J.A., Roberts A.B., Sangwan N., Skye S.M., Li L., Ho K.J., Varga J., DiDonato J.A., Tang W.W. (2020). Targeted Inhibition of Gut Microbial Trimethylamine N-Oxide Production Reduces Renal Tubulointerstitial Fibrosis and Functional Impairment in a Murine Model of Chronic Kidney Disease. Arterioscler. Thromb. Vasc. Biol..

[18] Tang W.H., Wang Z., Kennedy D.J., Wu Y., Buffa J.A., Agatisa-Boyle B., Li X.S., Levison B.S., Hazen S.L. (2015). Gut microbiota-dependent trimethylamine N-oxide (TMAO) pathway contributes to both development of renal insufficiency and mortality risk in chronic kidney disease. Circ. Res..

[19] Organ C.L., Otsuka H., Bhushan S., Wang Z., Bradley J., Trivedi R., Polhemus D.J., Tang W.W., Wu Y., Hazen S.L. (2016). Choline Diet and Its Gut Microbe-Derived Metabolite, Trimethylamine N-Oxide, Exacerbate Pressure Overload-Induced Heart Failure. Circ. Heart Fail..

[20] Li Z., Wu Z., Yan J., Liu H., Liu Q., Deng Y., Ou C., Chen M. (2019). Gut microbe-derived metabolite trimethylamine N-oxide induces cardiac hypertrophy and fibrosis. Lab. Investig..

[21] Organ C.L., Li Z., Sharp T.E., Polhemus D.J., Gupta N., Goodchild T.T., Tang W.H.W., Hazen S.L., Lefer D.J. (2020). Nonlethal Inhibition of Gut Microbial Trimethylamine N-oxide Production Improves Cardiac Function and Remodeling in a Murine Model of Heart Failure. J. Am. Heart Assoc..

[22] Yang W., Zhang S., Zhu J., Jiang H., Jia D., Ou T., Qi Z., Zou Y., Qian J., Sun A. (2019). Gut microbe-derived metabolite trimethylamine N-oxide accelerates fibroblast-myofibroblast differentiation and induces cardiac fibrosis. J. Mol. Cell Cardiol..

[23] Tang W.H., Wang Z., Levison B.S., Koeth R.A., Britt E.B., Fu X., Wu Y., Hazen S.L. (2013). Intestinal microbial metabolism of phosphatidylcholine and cardiovascular risk. N. Engl. J. Med..

[24] Tang W.H., Wang Z., Fan Y., Levison B., Hazen J.E., Donahue L.M., Wu Y., Hazen S.L. (2014). Prognostic value of elevated levels of intestinal microbe-generated metabolite trimethylamine-N-oxide in patients with heart failure: Refining the gut hypothesis. J. Am. Coll. Cardiol..

[25] Stec A., Maciejewska M., Paralusz-Stec K., Michalska M., Giebułtowicz J., Rudnicka L., Sikora M. (2023). The Gut Microbial Metabolite Trimethylamine N-Oxide is Linked to Specific Complications of Systemic Sclerosis. J. Inflamm. Res..

[26] Ernster V. (1994). Nested case-control studies. Prev. Med..

[27] Richardson C., Agrawal R., Lee J., Almagor O., Nelson R., Varga J., Cuttica M.J., Dematte J.D., Chang R.W., Hinchcliff M.E. (2016). Esophageal dilatation and interstitial lung disease in systemic sclerosis: A cross-sectional study. Semin. Arthritis Rheum..

[28] van den Hoogen F., Khanna D., Fransen J., Johnson S.R., Baron M., Tyndall A., Matucci-Cerinic M., Naden R.P., Medsger T.A., Carreira P.E. (2013). 2013 classification criteria for systemic sclerosis: An American college of rheumatology/European league against rheumatism collaborative initiative. Ann. Rheum. Dis..

[29] Wang Z., Klipfell E., Bennett B.J., Koeth R., Levison B.S., DuGar B., Feldstein A.E., Britt E.B., Fu X., Chung Y.-M. (2011). Gut flora metabolism of phosphatidylcholine promotes cardiovascular disease. Nature.

[30] Wang Z., Nicholls S.J., Rodriguez E.R., Kummu O., Hörkkö S., Barnard J., Reynolds W.F., Topol E.J., A DiDonato J., Hazen S.L. (2007). Protein carbamylation links inflammation, smoking, uremia and atherogenesis. Nat. Med..

[31] Romano K.A., Nemet I., Saha P.P., Haghikia A., Li X.S., Mohan M.L., Lovano B., Castel L., Witkowski M., Buffa J.A. (2023). Gut Microbiota-Generated Phenylacetylglutamine. Circ. Heart Fail..

[32] Huang Y., DiDonato J.A., Levison B.S., Schmitt D., Li L., Wu Y., Buffa J., Kim T., Gerstenecker G.S., Gu X. (2014). An abundant dysfunctional apolipoprotein A1 in human atheroma. Nat. Med..

[33] Wei R., Ni Y., Bazeley P., Grandhi S., Wang J., Li S.T., Hazen S.L., Tang W.H.W., LaFramboise T. (2021). Mitochondrial DNA Content Is Linked to Cardiovascular Disease Patient Phenotypes. J. Am. Heart Assoc..

[34] Senthong V., Li X.S., Hudec T., Coughlin J., Wu Y., Levison B., Wang Z., Hazen S.L., Tang W.W. (2016). Plasma Trimethylamine N-Oxide, a Gut Microbe-Generated Phosphatidylcholine Metabolite, Is Associated with Atherosclerotic Burden. J. Am. Coll. Cardiol..

[35] Wang Z., Levison B.S., Hazen J.E., Donahue L., Li X.M., Hazen S.L. (2014). Measurement of trimethylamine-N-oxide by stable isotope dilution liquid chromatography tandem mass spectrometry. Anal. Biochem..

[36] Jang J.W., Capaldi E., Smith T., Verma P., Varga J., Ho K.J. (2024). Trimethylamine N-oxide: A meta-organismal axis linking the gut and fibrosis. Mol. Med..

[37] Hausmann A.J., McMahan Z.H., Volkmann E.R. (2024). Understanding the gastrointestinal microbiome in systemic sclerosis: Methodological advancements and emerging research. Curr. Opin. Rheumatol..

[38] Wei J., Bhattacharyya Toutellotee W.G., Varga J. (2011). Emerging concepts and implications for targeted therapy. Autoimmun. Rev..

[39] Randrianarisoa E., Lehn-Stefan A., Wang X., Hoene M., Peter A., Heinzmann S.S., Zhao X., Königsrainer I., Königsrainer A., Balletshofer B. (2016). Relationship of Serum Trimethylamine N-Oxide (TMAO) Levels with early Atherosclerosis in Humans. Sci. Rep..

[40] Garcia-Perez I., Villasenor A., Wijeyesekera A., Posma J.M., Jiang Z., Stamler J., Aronson P., Unwin R., Barbas C., Elliott P. (2012). Urinary metabolic phenotyping the slc26a6 (chloride-oxalate exchanger) null mouse model. J. Proteome Res..

[41] Gavaghan McKee C.L., Wilson I.D., Nicholson J.K. (2006). Metabolic phenotyping of nude and normal (Alpk:ApfCD, C57BL10J) mice. J. Proteome Res..

[42] Stanley E.G., Bailey N.J., Bollard M.E., Haselden J., Waterfield C., Holmes E., Nicholson J. (2005). Sexual dimorphism in urinary metabolite profiles of Han Wistar rats revealed by nuclear-magnetic-resonance-based metabonomics. Anal. Biochem..

[43] Tang W.H., Wang Z., Shrestha K., Borowski A.G., Wu Y., Troughton R.W., Klein A.L., Hazen S.L. (2015). Intestinal microbiota-dependent phosphatidylcholine metabolites, diastolic dysfunction, and adverse clinical outcomes in chronic systolic heart failure. J. Card. Fail..

[44] Chen K., Zheng X., Feng M., Li D., Zhang H. (2017). Gut Microbiota-Dependent Metabolite Trimethylamine N-Oxide Contributes to Cardiac Dysfunction in Western Diet-Induced Obese Mice. Front. Physiol..

[45] Mihuta M.S., Paul C., Borlea A., Roi C.M., Pescari D., Velea-Barta O.-A., Mozos I., Stoian D. (2023). Connections between serum Trimethylamine N-Oxide (TMAO), a gut-derived metabolite, and vascular biomarkers evaluating arterial stiffness and subclinical atherosclerosis in children with obesity. Front. Endocrinol..

[46] Barrea L., Annunziata G., Muscogiuri G., Di Somma C., Laudisio D., Maisto M., De Alteriis G., Tenore G.C., Colao A., Savastano S. (2018). Trimethylamine-N-oxide (TMAO) as Novel Potential Biomarker of Early Predictors of Metabolic Syndrome. Nutrients..

[47] Lemaitre R.N., Jensen P.N., Wang Z.A., Fretts A.M., McKnight B., Nemet I., Biggs M.L., Sotoodehnia N., de Oliveira Otto M.C., Psaty B.M. (2021). Association of Trimethylamine N-Oxide and Related Metabolites in Plasma and Incident Type 2 Diabetes: The Cardiovascular Health Study. JAMA Netw. Open.

[48] Shimizu M., Cashman J.R., Yamazaki H. (2007). Transient trimethylaminuria related to menstruation. BMC Med. Genet..

[49] Rebouche C.J., Seim H. (1998). Carnitine metabolism and its regulation in microorganisms and mammals. Annu. Rev. Nutr..

[50] Barrea L., Muscogiuri G., Pugliese G., Graziadio C., Maisto M., Pivari F., Falco A., Tenore G.C., Colao A., Savastano S. (2021). Association of the Chronotype Score with Circulating Trimethylamine N-Oxide (TMAO) Concentrations. Nutrients.

[51] Kuhn T., Rohrmann S., Sookthai D., Johnson T., Katzke V., Kaaks R., von Eckardstein A., Müller D. (2017). Intra-individual variation of plasma trimethylamine-N-oxide (TMAO), betaine and choline over 1 year. Clin. Chem. Lab. Med. (CCLM).

