# Supplementary material for: Elevated Circulating Levels of Gut Microbe-Derived Trimethylamine N-Oxide Are Associated with Systemic Sclerosis
Source: J Clin Med. 2024 Oct 8;13(19):5984. doi: 10.3390/jcm13195984 (PMC11477889; doi:10.3390/jcm13195984)
Supplement: Supplementary file 1 [file jcm-13-05984-s001.zip › jcm-3201383-supplementary.pdf]

Supplemental Figure S1. Comparative analysis of serum TMAO concentration based on SSc group (A), SSc group and obesity (B), and SSc group and sex (C). For each box blot, the natural log of TMAO is shown on the Y-axis.

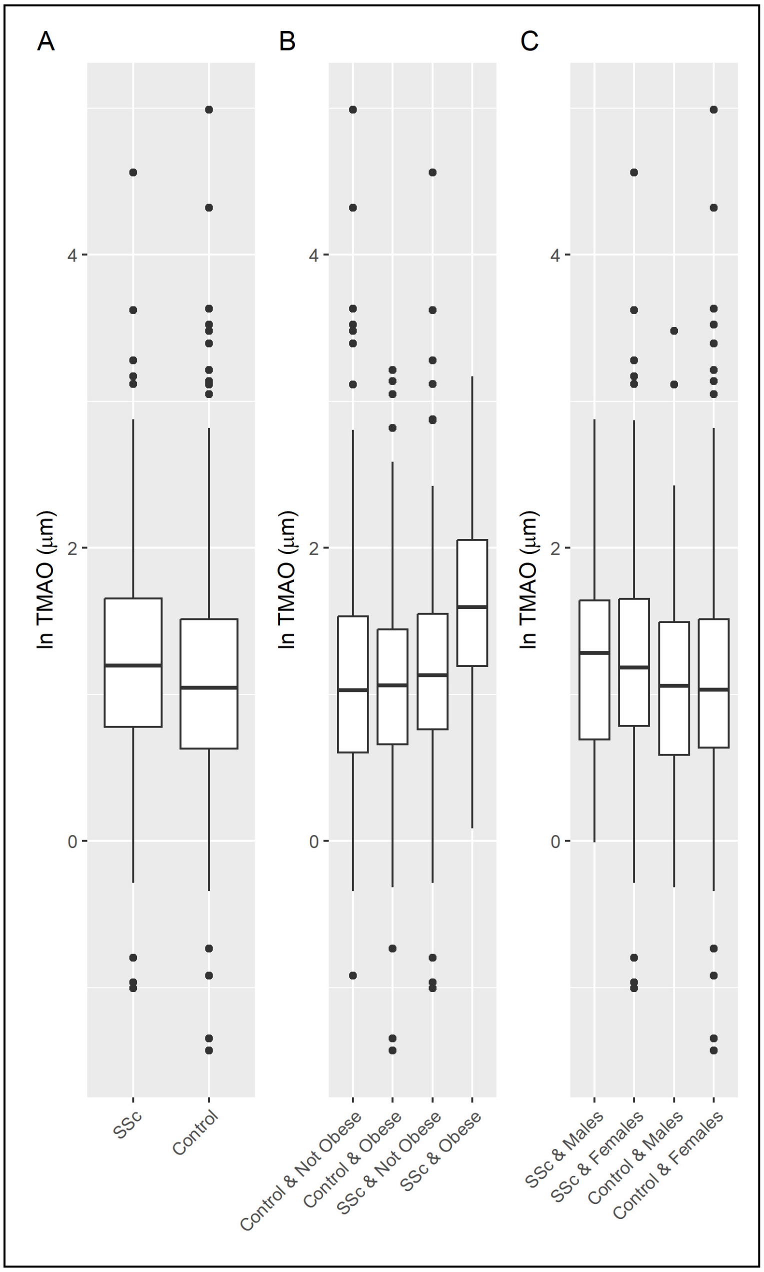

Supplemental Table S1.

A. Quantile Regression Parameter Estimates at the 25th Quantile of TMAO

	**Coefficient (SE)**	**95% CI**	***p* value**
Sex: Male vs. Female	0.427 (0.231)	(0.115, 0.637)	0.066
Group: SSc vs Control	0.450 (0.210)	(0.175, 0.631)	0.033*
BMI	0.015 (0.015)	(0.003, 0.033)	0.316
Age	0.013 (0.009)	(0.002,0.020)	0.122
Caucasian: Yes vs No	-0.281 (0.278)	(-0.396, 0.214)	0.940
eGFR	-0.021 (0.006)	(-0.033, -0.015)	0.001*

* Indicates *p* value < 0.05. In the quantile regression model TMAO is the outcome. Covariates in the quantile regression model are sex, group, BMI, age, race, and eGFR. SE, Standard Error; CI, Confidence Interval

B. Quantile Regression Parameter Estimates at the 50th Quantile of TMAO

	**Coefficient (SE)**	**95% CI**	***p* value**
Sex: Male vs. Female	0.158 (0.281)	(-0.130, 0.607)	0.575
Group: SSc vs Control	0.546 (0.251)	(0.357, 0.878)	0.030*
BMI	0.028 (0.018)	(0.004, 0.047)	0.232
Age	0.018 (0.012)	(-0.002, 0.030)	0.115
Caucasian: Yes vs No	0.039 (0.319)	(-0.215, 0.245)	0.902
eGFR	-0.032 (0.009)	(-0.046, -0.025)	<0.001*

* Indicates *p* value < 0.05. In the quantile regression model TMAO is the outcome. Covariates in the quantile regression model are sex, group, BMI, age, race, and eGFR. SE, Standard Error; CI, Confidence Interval

C. Quantile Regression Parameter Estimates at the 75th Quantile of TMAO

	**Coefficient (SE)**	**95% CI**	***p* value**
Sex: Male vs. Female	0.569 (0.437)	(-0.265, 1.251)	0.193
Group: SSc vs Control	0.785 (0.357)	(0.272, 1.411)	0.028*
BMI	0.005 (0.024)	(-0.013, 0.085)	0.836
Age	0.006 (0.020)	(-0.019, 0.034)	0.780
Caucasian: Yes vs No	-0.333 (0.522)	(-1.200, 0.794)	0.524
eGFR	-0.070 (0.014)	(-0.090, -0.042)	<0.001*

* Indicates p value < 0.05. In the quantile regression model TMAO is the outcome. Covariates in the quantile regression model are sex, group, BMI, age, race, and eGFR. SE, Standard Error; CI, Confidence Interval

Supplemental Table S2.

A. Spearman's Rho Correlations for metabolites and disease duration at baseline and progression over 1 year.

**Metabolites (μM)**	**Disease Duration or Progression Measures**	**Spearman's Rho Correlation**	***p* value***
TMAO	MRSS at Baseline	0.018	0.719
Choline	MRSS at Baseline	0.067	0.719
Betaine	MRSS at Baseline	0.039	0.719
Carnitine	MRSS at Baseline	-0.037	0.168
Butyrobetaine	MRSS at Baseline	-0.155	0.719
Crotonobetaine	MRSS at Baseline	0.040	0.981
TMAO	FVC at Baseline	-0.043	0.981
Choline	FVC at Baseline	-0.002	0.981
Betaine	FVC at Baseline	-0.027	0.981
Carnitine	FVC at Baseline	-0.025	0.096
Butyrobetaine	FVC at Baseline	0.170	0.981
Crotonobetaine	FVC at Baseline	0.003	0.210
TMAO	DLCO at Baseline	-0.153	0.633
Choline	DLCO at Baseline	-0.058	0.877
Betaine	DLCO at Baseline	0.020	0.344
Carnitine	DLCO at Baseline	-0.103	0.877
Butyrobetaine	DLCO at Baseline	0.011	0.344
Crotonobetaine	DLCO at Baseline	-0.099	0.271
TMAO	Disease Duration	0.095	0.829
Choline	Disease Duration	-0.015	0.719

Betaine	Disease Duration	0.100	0.271
Carnitine	Disease Duration	0.152	0.192
Butyrobetaine	Disease Duration	0.087	0.271
Crotonobetaine	Disease Duration	0.086	0.271
TMAO	Change in MRSS over 1 year	0.078	0.774
Choline	Change in MRSS over 1 year	-0.046	0.774
Betaine	Change in MRSS over 1 year	-0.031	0.774
Carnitine	Change in MRSS over 1 year	0.056	0.774
Butyrobetaine	Change in MRSS over 1 year	0.042	0.774
Crotonobetaine	Change in MRSS over 1 year	0.020	0.774
TMAO	Change in FVC over 1 year	0.031	0.806
Choline	Change in FVC over 1 year	0.011	0.879
Betaine	Change in FVC over 1 year	-0.134	0.360
Carnitine	Change in FVC over 1 year	-0.075	0.806
Butyrobetaine	Change in FVC over 1 year	-0.030	0.806
Crotonobetaine	Change in FVC over 1 year	0.036	0.806
TMAO	Change in DLCO over 1 year	0.164	0.099
Choline	Change in DLCO over 1 year	0.015	0.957
Betaine	Change in DLCO over 1 year	-0.032	0.957
Carnitine	Change in DLCO over 1 year	0.004	0.957
Butyrobetaine	Change in DLCO over 1 year	0.058	0.864
Crotonobetaine	Change in DLCO over 1 year	0.157	0.099

*Indicates *p* values are adjusted using the Benjamini-Hochberg procedure

B. Kruskal-Wallis Test for Comparing SSc Classification by Metabolites.

	**Limited cutaneous**<br><br>**N = 87**	**Diffuse cutaneous**<br><br>**N = 109**	***p***<br><br>**value***
TMAO, μM (median [IQR])	3.43 [2.23, 5.93]	3.21 [2.19, 5.01]	0.816
Choline (median [IQR])	38.03 [27.05, 44.49]	37.54 [30.24, 47.33]	0.816
Betaine (median [IQR])	36.36 [31.54, 41.51]	37.41 [31.00, 41.77]	1.000
Carnitine (median [IQR])	0.90 [0.79, 1.10]	0.89 [0.73, 1.07]	0.816
Butyrobetaine (median [IQR])	0.02 [0.01, 0.02]	0.02 [0.01, 0.02]	1.000
Crotonobetaine (median [IQR])	12.43 [10.36, 14.55]	13.88 [11.17, 16.18]	0.144

*Indicates *p* values are adjusted using the Benjamini-Hochberg procedure

C. Wilcoxon Rank Sum Test for Comparing Anti-Scl-70 (Anti-Topoisomerase-1 antibody) by Metabolites.

	**Negative**<br><br>**N = 149**	**Positive**<br><br>**N = 43**	***p***<br><br>**value***
TMAO, μM (median [IQR])	3.42 [2.23, 5.37]	2.94 [1.88, 4.55]	0.201
Choline, μM (median [IQR])	13.50 [11.01, 16.02]	12.53 [10.12, 14.00]	0.222
Betaine, μM (median [IQR])	38.85 [30.35, 46.28]	32.56 [26.55, 42.01]	0.201
Carnitine, μM (median [IQR])	37.12 [31.89, 41.88]	35.33 [29.37, 41.38]	0.374
Butyrobetaine, μM (median [IQR])	0.90 [0.77, 1.10]	0.91 [0.74, 1.04]	0.463
Crotonobetaine, μM (median [IQR])	0.02 [0.01, 0.03]	0.02 [0.01, 0.02]	0.255

*Indicates *p* values are adjusted using the Benjamini-Hochberg procedure

D. Wilcoxon Rank Sum Test for Anti-Neutrophil Antibody by Metabolites.

	**Negative**<br><br>**N=7**	**Positive**<br><br>**N=190**	***p* value***
TMAO (median [IQR])	4.48 [3.34, 4.98]	3.28 [2.18, 5.20]	0.781

Choline (median [IQR])	13.63 [11.24, 16.78]	13.16 [10.76, 15.72]	0.781
Betaine (median [IQR])	37.30 [32.10, 51.96]	37.74 [29.28, 45.39]	0.781
Carnitine (median [IQR])	36.70 [32.09, 41.24]	36.98 [30.96, 41.70]	0.893
Butyrobetaine (median [IQR])	0.87 [0.61, 1.04]	0.90 [0.77, 1.08]	0.781
Crotonobetaine (median [IQR])	0.02 [0.02, 0.03]	0.02 [0.01, 0.02]	0.720

*Indicates *p* values are adjusted using the Benjamini-Hochberg procedure

E. Wilcoxon Rank Sum Test for Anti-Centomere Antibody by Metabolites.

	**Negative**<br>**N=155**	**Positive**<br>**N=39**	***p***<br>**value***
TMAO (median [IQR])	3.15 [2.15, 4.99]	3.64 [2.45, 6.60]	0.252
Choline (median [IQR])	13.26 [10.85, 15.62]	12.37 [10.61, 16.28]	0.710
Betaine (median [IQR])	37.38 [28.44, 45.30]	38.16 [31.22, 45.18]	0.538
Carnitine (median [IQR])	36.38 [30.92, 41.68]	38.40 [32.98, 42.06]	0.538
Butyrobetaine (median [IQR])	0.88 [0.75, 1.07]	0.98 [0.84, 1.10]	0.282
Crotonobetaine (median [IQR])	0.02 [0.01, 0.02]	0.02 [0.01, 0.02]	0.282

*Indicates *p* values are adjusted using the Benjamini-Hochberg procedure

F. Wilcoxon Rank Sum Test for Anti-RNA Polymerase III by Metabolites.

	**Negative**<br>**N=108**	**Positive**<br>**N=56**	***p***<br>**value***
TMAO (median [IQR])	3.35 [2.21, 4.96]	3.54 [2.25, 5.73]	0.659
Choline (median [IQR])	12.48 [10.71, 15.28]	14.22 [11.98, 16.06]	0.126
Betaine (median [IQR])	36.06 [29.15, 44.80]	37.93 [29.38, 46.04]	0.920
Carnitine (median [IQR])	34.99 [29.41, 40.85]	37.35 [31.73, 41.10]	0.608
Butyrobetaine (median [IQR])	0.88 [0.74, 1.07]	0.88 [0.76, 1.05]	0.920
Crotonobetaine (median [IQR])	0.02 [0.01, 0.02]	0.02 [0.01, 0.03]	0.608

*Indicates *p* values are adjusted using the Benjamini-Hochberg procedure

G. Wilcoxon Rank Sum Test for Pulmonary Hypertension/Interstitial Lung Disease by Metabolites.

	**Negative** **N=69**	**Positive** **N=131**	***p* value***
TMAO, µM (median [IQR])	3.22 [2.15, 5.01]	3.38 [2.19, 5.27]	0.911
Choline, µM (median [IQR])	13.26 [10.72, 15.41]	13.18 [11.02, 15.91]	0.995
Betaine, µM (median [IQR])	38.16 [28.15, 44.67]	37.38 [29.51, 46.12]	0.995
Carnitine, µM (median [IQR])	38.39 [31.26, 43.39]	36.70 [30.97, 41.03]	0.911
Butyrobetaine, µM (median [IQR])	0.91 [0.81, 1.09]	0.88 [0.74, 1.08]	0.911
Crotonobetaine, µM (median [IQR])	0.02 [0.01, 0.03]	0.02 [0.01, 0.02]	0.911

*Indicates *p* values are adjusted using the Benjamini-Hochberg procedure

Supplemental Table S3.

Characteristics and metabolites of the 2 patient groups identified by hierarchical clustering. *P* values for the Wilcoxon rank sum test are reported for continuous variables. Chi square test or Fisher's exact test *p* values are reported for categorical variables.

	**Group 1** **N = 92**	**Group 2** **N = 108**	***p* value**
Age, years (median [IQR])	54.00 [45.75, 60.00]	51.00 [42.75, 60.00]	0.170
Female (%)	76 (82.6)	93 (86.1)	0.627
Caucasian (%)	75 (81.5)	90 (83.3)	0.881
BMI, kg/m² (median [IQR])	24.38 [21.03, 28.05]	25.14 [21.61, 28.69]	0.510
eGFR, mL/min/1.73 m^2 (median [IQR])	91.91 [73.95, 106.07]	93.10 [76.07, 105.38]	0.845
Creatinine, mg/dL (median [IQR])	0.78 [0.68, 0.90]	0.76 [0.68, 0.88]	0.600
History of Cardiovascular Disease (%),	5 (5.7)	1 (1.0)	0.099
History of Diabetes Mellitus (%)	2 (2.3)	3 (3.0)	1.000
Metabolites			
TMAO, µm (median [IQR])	3.39 [2.28, 4.98]	3.11 [2.12, 5.87]	0.724
Choline, µm (median [IQR])	13.40 [11.17, 15.46]	13.05 [10.46, 15.56]	0.388
Betaine, µm (median [IQR])	40.44 [34.64, 45.80]	31.66 [24.39, 45.52]	0.001*
Carnitine, µm (median [IQR])	37.77 [33.39, 42.86]	36.06 [29.41, 40.78]	0.033*
Butyrobetaine, µm (median [IQR])	0.93 [0.81, 1.12]	0.87 [0.74, 1.03]	0.088
Crotonobetaine, µm (median [IQR])	0.02 [0.01, 0.03]	0.02 [0.01, 0.02]	0.821
Disease Duration, months (median [IQR])	47.50 [17.00, 96.75]	44.00 [18.50, 95.00]	0.929
Early Disease Duration (≤ 24 months) (%)	29 (31.5)	31 (29.0)	0.814
SSc Diagnosis (%)			0.352
Limited cutaneous	35 (38.0)	52 (48.1)	
Diffuse cutaneous	55 (59.8)	54 (50.0)	

SSS	2 (2.2)	2 (1.9)	
Autoantibodies			
Antinuclear antibody (ANA)(%)	87 (96.7)	103 (96.3)	1.000
Anticentromere antibody (ACA)(%)	13 (14.1)	26 (24.1)	0.209
Antitopoisomerase I antibody (%)	16 (18.2)	27 (26.0)	0.265
Anti-RNA Polymerase III Antibody (%)	30 (32.6)	26 (24.1)	0.160
Skin Thickening (%)	79 (85.9)	94 (87.0)	0.970
Active Digital Ulcer (%)	10 (11.4)	13 (12.9)	0.926
Ulcers/Pitting Scars (%)			0.004*
Ulcer	12 (13.0)	23 (21.3)	
Pitting Scars	24 (26.1)	45 (41.7)	
Negative	56 (60.9)	40 (37.0)	
Telangiectasias (%)	62 (67.4)	87 (80.6)	0.049*
Abnormal Nailfold Capillaries** (%)	57 (62.0)	65 (60.2)	0.912
PAH or ILD (%)	54 (58.7)	77 (71.3)	0.086
Raynaud’s Phenomenon (%)	92 (100.0)	106 (98.1)	0.549
Antibodies (%)	58 (63.0)	76 (70.4)	0.343
FVC Baseline, liters (median [IQR])	82.50 [66.75, 93.00]	81.00 [70.25, 89.00]	0.890
Baseline DLCO, ml/mm Hg/min (median [IQR])	68.23 [49.41, 78.11]	64.00 [52.88, 77.24]	0.740
Baseline MRSS (median [IQR])	7.50 [4.00, 16.25]	8.00 [4.00, 18.25]	0.874
Change in Measures at 1 Year			
FVC Change, liters (median [IQR])	1.00 [-4.50, 5.25]	0.00 [-6.00, 6.00]	0.837
DLCO Change, ml/mmhg/min (median [IQR])	0.18 [-6.16, 5.57]	-1.36 [-6.54, 5.13]	0.416

MRSS Change (median [IQR])	0.00 [-4.00, 0.00]	0.00 [-2.25, 0.00]	0.189
MRSS Change ≤ - 5 (%)	21 (22.8)	20 (18.5)	0.601
MRSS Change ≥ 5 (%)	5 (5.4)	9 (8.3)	0.580

*Indicates ***p*** value < 0.05. **Microscopic evaluation of nailfold capillaries were scored as “normal” or “abnormal” morphologic patterns. BMI, body mass index; DLCO, diffusing capacity of the lungs for carbon monoxide; eGFR, estimated glomerular filtration rate; FVC, forced vital capacity; IQR, interquartile range; ILD, interstitial lung disease; MRSS, modified Rodnan skin score; N, number; PAH, pulmonary arterial hypertension; SSc, systemic sclerosis; TMAO, trimethylamine N-oxide.
